# The Impact of Ambient Air Pollution on Allergic Rhinitis Symptoms: A Prospective Follow-Up Study

**DOI:** 10.3390/toxics12090663

**Published:** 2024-09-11

**Authors:** Wen Sun, Chan Ding, Zhuoying Jiang, Xinliang Zheng, Jinlan Jiang, Huadong Xu

**Affiliations:** 1 School of Public Health, Hangzhou Medical College, 182 Tianmushan Road, Xihu District, Hangzhou 310013, China; 881012022125@hmc.edu.cn (W.S.); 130232023252@hmc.edu.cn (C.D.); jiangzhuoying0304@163.com (Z.J.); zxl2695291160@163.com (X.Z.); 2 The Second School of Clinical Medicine, Zhejiang Chinese Medical University, 548 Binwen Road, Binjiang District, Hangzhou 310053, China

**Keywords:** allergic rhinitis, ambient air pollution, prospective study, nasal symptoms

## Abstract

Air pollution has become a serious public health problem and there is evidence that air pollution affects the incidence of allergic rhinitis. To further investigate the effect of ambient air pollutants on the severity of allergic rhinitis symptoms, a prospective follow-up study in patients with allergic rhinitis was conducted. A total of 167 allergic rhinitis patients with a mean age of 35.4 years, who were visiting the hospital, were enrolled. The daily symptom severity of allergic rhinitis and the concentrations of six air pollutants, including PM_2.5_, PM_10_, SO_2_, CO, O_3_ and NO_2,_ were collected through follow-up investigations. The impact of ambient air pollutants on symptom severity was assessed via multi-pollutant models. Among several typical ambient air pollutants, we observed correlations of allergic rhinitis symptoms with PM_2.5_, PM_10_, CO, SO_2_ and NO_2_, whereas O_3_ showed no such correlation. Specifically, PM_2.5_ and PM_10_ were significantly associated with sneezing and nasal blockage. NO_2_ was significantly correlated with symptoms of rhinorrhea, itchy nose and itchy eyes. CO was significantly linked to sneezing and nasal blockage symptoms. These air pollutants not only had a direct impact on allergic rhinitis symptoms but also exhibited a lagging effect. This study indicates that short-term exposure to air pollutants is associated with exacerbation of nasal symptoms in patients with allergic rhinitis, leading to a decline in their quality of life.

## 1. Introduction

Since the onset of global industrialization, the pollution problem has become increasingly serious. To date, air pollution remains a major public health problem. In fact, according to a report released by the World Health Organization (WHO) in 2022, 99% of the global population lived in places that failed to meet the air quality guidelines of the WHO [1]. It was further estimated that outdoor ambient air pollution contributed to 4.2 million premature deaths globally in 2019 [2]. Research on air pollution and respiratory health has been extensive, driven by the direct impact of air pollutants on the human respiratory system. Numerous studies have demonstrated associations between air pollution and health issues such as acute lower respiratory infections, chronic obstructive pulmonary disease (COPD), asthma, lung cancer and allergic rhinitis [3]. These studies also highlight that there is no identified threshold below which exposure to air pollution can be deemed safe [4,5].

Allergic rhinitis, one of the most prevalent chronic diseases globally, is typically characterized by sneezing, nasal itching, nasal blockage and runny nose. It affects an estimated 10–40% of the global population, annually contributing to approximately USD 17.5 billion in health-related economic losses [6]. For those afflicted, allergic rhinitis can cause sleep disturbances, fatigue, mood depression, reduced sense of smell and cognitive impairment, thereby profoundly impacting their quality of life and productivity in both learning and work settings [7,8]. Furthermore, allergic rhinitis stands as a notable risk factor for asthma, otitis media and allergic conjunctivitis. Prolonged exposure to allergic rhinitis can exacerbate or precipitate lower respiratory tract disorders, particularly asthma [9]. Allergic rhinitis is a multifactorial disease determined by a combination of genetics and the environment. In recent decades, the prevalence of allergic rhinitis among populations has risen steadily, with atmospheric pollution increasingly recognized as a pivotal environmental risk factor [10]. Epidemiological investigations have revealed that exposure to airborne fine particulate matter (PM_2.5_) was associated with heightened rates of clinic visits for allergic rhinitis [11]. Animal studies demonstrated that PM_2.5_ exposure exacerbated nasal allergy symptoms in an ovalbumin (OVA) sensitized murine model of allergic rhinitis [12].

The role of PM_2.5_ in allergic rhinitis has been extensively studied, revealing its capacity to induce cellular oxidative stress and inflammatory responses, particularly in inflamed nasal mucosal epithelial cells [11,13]. PM_2.5_-induced oxidative stress is postulated as a mechanism exacerbating allergic rhinitis symptoms [14]. An allergic rhinitis mouse model study demonstrated that *N*-acetylcysteine, as an antioxidant agent, alleviated PM_2.5_-induced symptoms, underscoring involvement of PM_2.5_ in oxidative stress and inflammation [14]. With advancing research, the epigenetic impacts of PM_2.5_ have been gaining attention in the last decade. Exposure to PM_2.5_ in the nasal mucosa of allergic rhinitis rats induced oxidative stress, which triggered autophagy through the damage of DNA, RNA and proteins. This process potentially exacerbated the condition by intensifying autophagy activity. Furthermore, they discovered that miR-338-3p effectively suppressed the autophagy induced by PM_2.5_ [15]. In an allergic rhinitis mouse model, it was shown that PM_2.5_ exposure led to the elevated DNA methylation of the IFN-γ gene promoter in CD4^+^ T cells, mediated by the ERK-DNMT pathway. This resulted in a reduction in Th1 cell numbers, a perpetuation of the Th1/Th2 imbalance and the exacerbation of allergic rhinitis [16].

Unlike the case with PM_2.5_, epidemiological inquiries into the relationship between sulfur dioxide (SO_2_) and allergic rhinitis have produced a heterogenous set of results: whereas certain studies have discerned a connection between SO_2_ exposure and the onset of allergic rhinitis [17], others fail to corroborate this association [18], thereby highlighting an inconclusive landscape. Besides variations in research methodologies, factors such as geographical differences, distinct time frames and the diversity of study populations have also contributed to the complexity of understanding how SO_2_ impacts allergic rhinitis. In an experiment utilizing an HDM-induced allergic rhinitis mouse model, brief exposure to SO_2_ not only augmented nasal symptom severity but also elevated serum Immunoglobulin E (IgE) levels and increased the infiltration of eosinophils in allergic rhinitis mice, alongside up-regulated expression of Th1/Th2/Th17 cytokines in the nasal mucosa. Strikingly, they found that prolonged SO_2_ exposure had the opposing effect of reducing serum IgE levels in these mice [19]. While numerous studies attest to the influence of ozone (O_3_), nitrogen dioxide (NO_2_), carbon monoxide (CO) and SO_2_ on the progression of allergic rhinitis, there is still a relative dearth of dedicated research in this area.

In the realm of epidemiological explorations of allergic rhinitis in relation to air pollution, numerous researchers have employed retrospective studies [20,21,22] as well as cohort study designs [23,24] to explore the relationships between ambient pollutant concentrations and outpatient-visit frequencies. Existing epidemiological and experimental research has illuminated the link between air pollution and allergic rhinitis, but many questions remain undetermined. One such question pertains to whether a discernible time-delay exists between pollutant exposure and allergic rhinitis symptoms. More attention has been paid to the effects of air pollutants on the incidence and prevalence of allergic rhinitis [25,26], whereas less environmental epidemiological research has been conducted on the effects of ambient air pollutants on the severity of allergic rhinitis symptoms—essentially, on the progression and exacerbation of the condition. These knowledge gaps limit our comprehensive understanding of disease mechanisms and hinder the development of precise prevention and treatment strategies.

In view of the above background, this study aimed to investigate in depth the dynamic relationship between ambient air pollution levels and allergic rhinitis symptoms through a prospective study, with a special focus on the effect on symptoms of fluctuations in pollutants. By integrating data from multiple sources, including air quality monitoring records and the medical records of allergic rhinitis patients, this study could provide new evidence to elucidate the impact of air pollution on allergic rhinitis.

## 2. Materials and Methods

### 2.1. Study Design and Participants

This panel study, conducted from May 2023 to January 2024, recruited patients with allergic rhinitis who visited the same hospital and had lived continuously in Hangzhou city, China, for at least one year. Ultimately, 167 participants agreed to and completed the research study, all of whom lived within 5 km of the hospital. The age range of the participants was around 35 years. Baseline investigations were conducted to gather the demographic details, the medical histories, the family histories and the lifestyle habits of the subjects, which were considered as control variables in the analysis. All included subjects were diagnosed with allergic rhinitis by professional physicians according to the guidelines for the Diagnosis and Treatment of Allergic Rhinitis in China [9]; these guidelines encompass recurrent symptoms such as sneezing, rhinorrhea, nasal blockage and nasal itching, alongside serological confirmation of specific IgE antibodies to allergens. Individuals with severe cardiopulmonary conditions, immune system disorders, those undergoing immunosuppressive therapies, pregnant or breastfeeding individuals and those with non-allergic rhinitis, nasal polyps, or chronic sinusitis were excluded from this study. During the entire study period, the allergic rhinitis symptoms were tracked daily through questionnaires (Table S1). All patients completed the survey and had complete symptom data. Ethical clearance for this research was obtained from the Ethics Committee of Hangzhou Medical College and written informed consent was secured from all participants prior to their enrollment in this study.

### 2.2. Measurement of Ambient Air Pollution Data

Atmospheric quality monitoring data were collected from the China National Environmental Monitoring Center (CNEMC, http://www.cnemc.cn/ (accessed on 24 April 2024)), as announced by the local environmental protection monitoring center. The data were obtained from the average daily air monitoring report in Hangzhou City, where the daily average concentration of ambient air pollutants is calculated as the arithmetic mean of the hourly concentrations over a 24 h period (O_3_ concentration value is the arithmetic mean of the ozone concentration over the largest consecutive 8 h period of the day). This study focused on the main air pollutants monitored in daily life, including PM_10_, PM_2.5_, O_3_, SO_2_, NO_2_ and CO. This study employed the daily average concentrations of these pollutants as reported by the monitoring center.

### 2.3. Health Effect Measurements

Throughout the follow-up period of this study, information on the allergic rhinitis symptoms of the study subjects was collected. In accordance with the Chinese Guideline for Allergic Rhinitis [9], symptoms included sneezing (the number of episodes of paroxysmal sneezing in a day), rhinorrhea (the number of episodes of nose blowing in a day), nasal blockage and nasal itching, as well as eye itching. The symptom scoring system ranged from a minimum of 0 to a maximum of 3. Details of the specific allergic rhinitis symptom scoring system are presented in Table S1.

### 2.4. Statistical Analysis

All data were expressed as mean ± standard deviation (SD) for the continuous variables or as percentages for the categorical variables. Spearman correlation analysis was used to investigate the correlation between the pollutants. Based on the previous literature [27], generalized linear mixed models (GLMMs) were employed to analyze the nasal symptom changes according to air pollutants among patients, incorporating each unique identifier of the study subject (as a random intercept model) to account for the repeated measurements of symptoms. Guided by the Akaike Information Criterion (AIC), this study exhaustively examined various pollutants and ultimately constructed models encompassing the six atmospheric pollutants, with stratification based on the included pollutants. Given the interdependence among levels of air pollutants, we compared each optimal multi-pollutant model based on the number of pollutants included. The formula for the GLMM is as follows:

Y_it_ = b_0_ + u_i_ + A_1_X_1_ + … + A_n_X_n_ + *β* Pollutants + ε_it_

where Y_it_ is the allergic rhinitis symptom score in the i-th subject at time t, b_0_ means the total intercept, u_i_ means the random intercept for the subject i, X_1_–X_n_ mean covariates, A_1_–A_n_ are regression coefficients for X_1_–X_n_, *β* means the regression coefficient for air pollutant and ε_it_ is the error for the i-th subject at time t.

The corresponding symptoms were represented by *β*-values, which can be positive or negative: positive values indicate an increase in the health effect with an increase in pollutant concentration, while negative values indicate a decrease in the health effect. When the 95% confidence intervals of the *β*-values include zero, there is no significant correlation between the level of exposure to the pollutant and the health outcome. All statistical analyses were conducted using R software version 4.4.1 (R Foundation for Statistical Computing) and a two-tailed *p*-value < 0.05 was deemed statistically significant.

## 3. Results

### 3.1. Participants

The baseline characteristics of the participants and their symptom scores at enrollment are presented in Table 1. A total of 167 participants were enrolled in this study, with a mean age of 35.4 years. In total, 60 of the participants (35.9%) were male and 107 (64.1%) were female. The mean body mass index (BMI) was 23.9 ± 3.9 kg/m^2^ for males and 21.1 ± 2.5 kg/m^2^ for females. The mean serum IgE level among participants was 376.5 IU/mL. Among the participants, 13 (7.8%) were smokers, while 154 (92.2%) were non-smokers. Additionally, 24 (14.4%) participants were alcohol consumers and 143 (85.6%) were non-drinkers. At the time of enrollment, the study participants exhibited symptom scores for allergic rhinitis as follows: sneezing scored 1.4 ± 0.8, nasal blockage scored 2.3 ± 0.8, rhinorrhea scored 1.7 ± 0.7, an itchy nose scored 1.5 ± 0.8 and itchy eyes scored 1.4 ± 0.9. These scores indicate that allergic rhinitis was indeed affecting the daily lives of the study subjects.

### 3.2. Ambient Air Pollution

**Figure 1:**
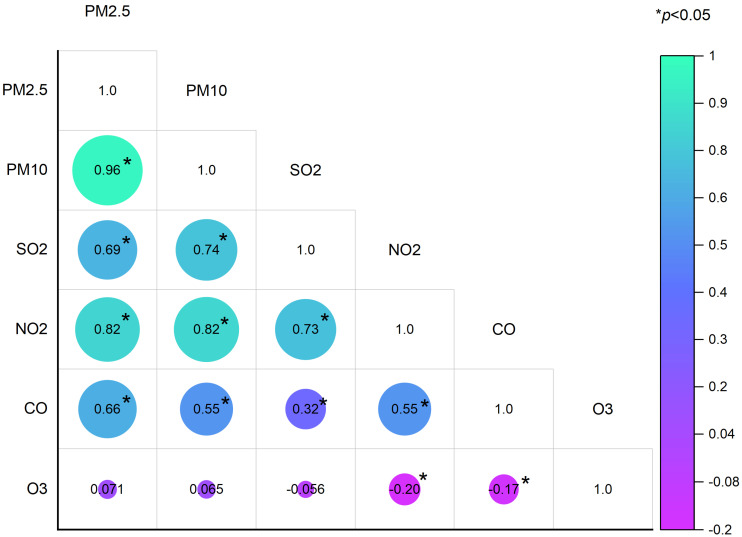
The correlation between the air pollutants on lag 0. The numbers of the figure are spearman correlation coefficients. PM_2.5_, particulate matter with diameter < 2.5 μm; PM_10_, particulate matter with diameter < 10 μm; SO_2_, sulfur dioxide; NO_2_, nitrogen dioxide; O_3_, ozone; CO, carbon monoxide.

Table 2 illustrates the daily average concentrations of ambient air pollutants on a monthly basis during the follow-up study period. Notably, PM_2.5_, PM_10_, SO_2_ and NO_2_ exhibited evident variations over the progression of months. Specifically, concentrations in the atmosphere demonstrated a gradual decline from May through July, followed by an upward trend from July until January of the following year. Figure 1 depicts the correlations among these air pollutants. These included significant correlations between PM_2.5_ and PM_10_ (*r* = 0.96), PM_2.5_ and SO_2_ (*r* = 0.69), PM_2.5_ and NO_2_ (*r* = 0.82), PM_2.5_ and CO (*r* = 0.66), PM_10_ and SO_2_ (*r* = 0.74), PM_10_ and NO_2_ (*r* = 0.82), PM_10_ and CO (*r* = 0.55), SO_2_ and NO_2_ (*r* = 0.73), SO_2_ and CO (*r* = 0.32), NO_2_ and CO (*r* = 0.55), NO_2_ and O_3_ (*r* = −0.20) and CO and O_3_ (*r* = −0.17).

### 3.3. Lagged Effects of Ambient Air Pollutants on Allergic Rhinitis Symptoms

**Figure 2:**
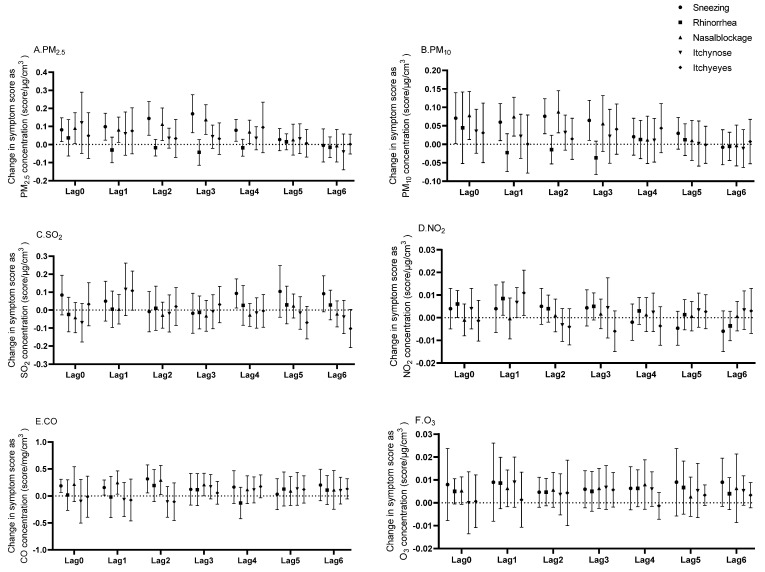
Lagged effects of air pollutants on allergic rhinitis symptom scores in patients with allergic rhinitis. Lagged effects of PM_2.5_ (**A**), PM_10_ (**B**), SO_2_ (**C**), NO_2_ (**D**), CO (**E**), and O_3_ (**F**) on allergic rhinitis symptoms are presented. PM_2.5_, particulate matter with diameter < 2.5 μm; PM_10_, particulate matter with diameter < 10 μm; SO_2_, sulfur dioxide; NO_2_, nitrogen dioxide; O_3_, ozone; CO, carbon monoxide.

The lagged effects of the ambient air pollutants on allergic rhinitis symptoms are shown in Figure 2 and Figure S1 and in Table S2. The symptom scores indicate the severity of the symptoms, with higher scores indicating greater severity (Table S1). The *β*-values represent the change in symptom scores for each unit increase in pollutant concentration. PM_2.5_ emerged as a prominent influencer, showing statistically significant links with both sneezing (*β* ranging from 0.072 to 0.141, with *p*-values between <0.001 and 0.041) and nasal blockage (*β* ranging from 0.068 to 0.123, *p*-values from 0.006 to 0.034) across lags 0 to 4, peaking in impact at lag 3 for sneezing (*β* = 0.141, *p* = 0.019) and nasal blockage (*β* = 0.123, *p* = 0.006). PM_10_ also demonstrated a significant association with sneezing across lags 0 to 3 (*β* ranging from 0.052 to 0.082, *p*-values from 0.009 to 0.045) and with nasal blockage from lags 0 to 2 (*β* ranging from 0.071 to 0.081, *p*-values from 0.002 to 0.024). NO_2_ exhibited significant correlations with rhinorrhea over lags 0 to 1 (*β* ranging from 0.006 to 0.008, *p*-values from 0.030 to 0.048). CO showed significant correlations with sneezing from lags 0 to 2 (*β* ranging from 0.122 to 0.283, *p*-values from 0.001 to 0.021), with the highest impact observed at lag 2 (*β* = 0.283, *p* = 0.001) and it was correlated with nasal blockage from lag 1 to lag 3 (*β* ranging from 0.101 to 0.236, *p*-values from 0.008 to 0.032), reaching its maximum effect on nasal blockage at lag 2 (*β* = 0.236, *p* = 0.008). SO_2_ was significantly correlated with sneezing specifically at lag 4 (*β* = 0.093, *p* = 0.025).

## 4. Discussion

This study investigated the association between allergic rhinitis symptoms and the levels of ambient air pollutants by tracking changes in the severity of rhinitis symptoms among 167 patients based on the panel design, concurrently monitoring fluctuations in pollutant concentrations during this timeframe. Our findings suggest that certain ambient air pollutants are not only correlated with allergic rhinitis symptoms, but they also show delayed or lagged effects.

In the WHO Global Air Quality Guidelines issued in December 2021 [1], the air quality guideline levels of PM_2.5_, PM_10_, O_3_, NO_2_, SO_2_ and CO were 15 μg/m^3^, 45 μg/m^3^, 100 μg/m^3^, 25 μg/m^3^, 40 μg/m^3^ and 4 μg/m^3^, respectively. During the study period, the concentrations of PM_2.5_, PM_10_ and NO_2_ were slightly below the guideline levels on some days, generally remaining at the same level. The levels of SO_2_ and CO were lower than the guideline levels throughout the study period. Therefore, our study has a certain degree of generalizability. Vehicle exhaust emissions, kerosene combustion, industrial emissions and biomass combustion are common sources of ambient air pollutants. Whereas NOx and CO are the main precursors of surface O_3_ [28], NOx and SO_2_ are important precursors of PM_2.5_ and their oxidized product NO_3_ may be the main driver of PM_2.5_ [29,30]. The correlation of PM with CO, SO_2_ and NO_2_ was also found in our study, which makes it difficult to isolate the effect of a single pollutant on allergic rhinitis symptoms. This intricate interplay among pollutants explains why the results of related epidemiological studies frequently show inconsistencies and have yet to be fully reconciled.

Considering the complex interactions and potential synergistic effects between pollutants, we used multi-pollutant modeling and found that PM_2.5_, PM_10_, NO_2_ and CO were each associated with allergic rhinitis symptoms. Specifically, our findings indicate that PM_2.5_, PM_10_ and CO cumulatively affect sneezing and nasal blockage, while NO_2_ shows a cumulative effect on rhinorrhea, itchy nose and itchy eyes. Consistent with our results, Tang et al. reported that short-term exposures to ambient air pollutants, including PM_2.5_, PM_10_ and CO, were linked to a heightened frequency of outpatient visits for allergic rhinitis [31]. Moreover, elevated outdoor concentrations of NO_2_ and PM_10_ were correlated with a higher likelihood of airway allergies and allergic rhinitis in children [32]. Luo and colleagues utilized Cox proportional hazard models to evaluate the relationship between long-term air pollutant exposure and allergic rhinitis risk, concluding that prolonged exposure to PM_2.5_, PM_10_ and NO_2_ elevated the likelihood of developing allergic rhinitis [33]. In alignment with our findings, a cohort investigation revealed a dose–response relationship between chronic air pollution exposure and rhinitis severity in adults. Furthermore, heightened levels of exposure to PM_2.5_, PM_10_ and NO_2_ were directly linked to intensified rhinitis symptoms [34]. Regarding SO_2_ and O_3_, a population-based study found a positive correlation between ambient SO_2_ levels and allergic rhinitis risk in primary school children [17]. Meanwhile, a strong association between prolonged environmental O_3_ exposure and an augmented allergic rhinitis symptom risk was reported in children [35]. Conversely, a time-series study reported that exposure to PM_10_, NO_2_ and O_3_ significantly elevated the hospitalization risk due to allergic rhinitis, whereas SO_2_ did not emerge as a significant risk factor for allergic rhinitis-related hospital admissions [18]. Intriguingly, despite SO_2_ and CO concentrations being well below air quality guideline levels during the study period, we still found a weak correlation between these pollutants and allergic rhinitis symptoms. The association between O_3_ and increased allergic rhinitis symptoms, however, was not significant. Differences in the results of similar studies might be attributed to variations in the study population, geographic location and timing. These factors can influence the levels and proportions of ambient air pollutants, as well as the age demographics of the study populations. Additionally, regional climate differences may also contribute to these discrepancies.

Regarding the lagged effects of air pollutants on allergic rhinitis, discrepancies in results across different regions, time periods and research methodologies have been observed. In this study, we found that PM_2.5_ was significantly associated with sneezing and nasal blockage symptoms from lag 0 to lag 4. A previous study reported that PM_2.5_ concentrations were associated with an increased risk of allergic rhinitis, occurring at lags 1 and 2, partly aligning with our findings [36]. In a study conducted in Lanzhou, China, short-term exposure to air pollutants was linked to a rise in allergic rhinitis visits, specifically with O_3_ influencing visits at a lag of 0–6 days and CO at 0–7 days [37]. Another study identified significant associations between CO and O_3_ exposure and respiratory outpatient visits across lags 0 to 4, but found no significant link between SO_2_ exposure and such visits. In addition, NO_2_ was relevant to respiratory outpatient visits only at lag 1 [38]. In this study, NO_2_ showed a significant correlation with rhinorrhea symptoms at lags 0 to 1, indicating an escalating trend. It also correlated with itchy nose and eye symptoms at lag 1. CO was significantly correlated with nasal symptoms from lag 0 to lag 2, while SO_2_ was found to have a significant correlation with sneezing symptoms at lag 4. In addition to aligning with previous research findings, the significance of our study lies in its extension beyond the scope of earlier works, which primarily focused on the incidence of allergic rhinitis and hospital visits. Our research narrows the gap by delving into a less-explored realm of nasal allergy symptoms.

In this study, we innovatively employed a panel research methodology to investigate the impact of ambient air pollutants on symptoms of allergic rhinitis, thereby enhancing the credibility of our results and refining our understanding of how individual pollutants affect several typical symptoms of the disease. Nonetheless, it is important to acknowledge its limitations. Our sample solely comprised patients from a single hospital, numbering only 167 individuals, which may limit the generalizability. Additionally, unlike clinical trials, ecological studies cannot directly measure individual exposure levels to ambient air pollutants. For descriptive studies on air pollutants, we used data from atmospheric monitoring stations and applied GLMMs to assess relationships between exposure levels and symptoms. This approach might lead to less accurate study results due to the indirect estimation of exposure. Allergens may have a correlation with pollutants that can also have an effect on allergic rhinitis symptoms. There is no way for this study to assess daily, individual-specific allergen exposure levels. Although allergens may act as a confounding factor, potentially affecting the results, this is a common limitation of ecological research. Similarly, the fact that our survey did not explore the effects of temperature and humidity on the study subjects makes it impossible to determine the effects of temperature and humidity on the results, which is also a limitation of this paper. We plan to recruit more patients to further investigate the impact of air pollution on different types of allergic rhinitis.

## 5. Conclusions

This study employed a prospective follow-up design to investigate the association between a mixture of various ambient air pollutants and the symptoms of allergic rhinitis in patients. This study found that ambient air pollutants were correlated with the severity of allergic rhinitis symptoms, with air pollutants exhibiting delayed effects in the multi-pollutant model. This finding thereby added to the evidence regarding the associations between short-term concurrent exposure to air pollutants and allergic rhinitis symptoms.

## Figures and Tables

**Table 1:** Baseline characteristics of the study population (*N* = 167).

Variable	Mean ± SD or *N* (%)
Age (years)	35.4 ± 12.1
Gender	
Male	60 (35.9%)
Female	107 (64.1%)
High (cm)	
Male	175 ± 5.2
Female	160.9 ± 5.6
Weight (kg)	
Male	73.4 ± 12.7
Female	55 ± 7.3
BMI (kg/m^2^)	
Male	23.9 ± 3.9
Female	21.1 ± 2.5
IgE (IU/mL)	376.5 ± 398
Smoking status	
Smoker	13 (7.8%)
Never smoker	154 (92.2%)
Drinking status	
Drinker	24 (14.4%)
Never drinker	143 (85.6%)
Sneezing ^a^	1.4 ± 0.8
Nasal blockage ^a^	2.3 ± 0.8
Rhinorrhea ^a^	1.7 ± 0.7
Itchy nose ^a^	1.5 + 0.8
Itchy eyes ^a^	1.4 ± 0.9

^a^ The symptom scores at enrollment.

**Table 2:** The means of the ambient air pollutants by month during the follow-up study period.

	May	June	July	August	September	October	November	December	January
PM_2.5_ (μg/m^3^) ^a^	27.6 ± 9.8	23.2 ± 9.4	14.4 ± 5.4	19.1 ± 7.7	22.1 ± 9.3	29.7 ± 15.3	34.5 ± 13.6	53.4 ± 37.1	66.8 ± 32
PM_10_ (μg/m^3^) ^a^	49.6 ± 18.1	39.2 ± 15.4	25.8 ± 7.9	31.8 ± 10.9	34 ± 12.9	47.3 ± 19.7	63.1 ± 22.1	81.1 ± 50.8	93 ± 42.4
SO_2_ (μg/m^3^) ^a^	6.2 ± 0.8	5.9 ± 0.8	5.6 ± 0.5	5.7 ± 0.4	5.9 ± 0.6	7.1 ± 0.6	7.3 ± 1	7.5 ± 1.2	7.6 ± 1.3
NO_2_ (μg/m^3^) ^a^	26 ± 6.3	22.2 ± 4.4	16 ± 3.7	16.6 ± 3.3	19.5 ± 3.2	32.4 ± 11.2	41.3 ± 12.2	49.2 ± 21.2	47.3 ± 14.7
CO (mg/m^3^) ^a^	0.6 ± 0.1	0.6 ± 0.1	0.6 ± 0.1	0.6 ± 0.1	0.7 ± 0.1	0.6 ± 0.1	0.7 ± 0.1	0.8 ± 0.2	0.9 ± 0.2
O_3_ (μg/m^3^) ^b^	128.7 ± 37.5	131.9 ± 57.6	96.1 ± 33.5	130.5 ± 45.7	123.9 ± 43.8	126.5 ± 29.7	77.6 ± 27.9	51 ± 22.8	57.5 ± 21.4

^a^ The arithmetic means of the daily average concentrations. ^b^ The arithmetic means of the daily maximum continuous 8 h concentrations.

## Data Availability

The data of this study are available upon reasonable request to the corresponding authors.

## Supplementary Materials

The following supporting information can be downloaded at https://www.mdpi.com/article/10.3390/toxics12090663/s1. Table S1: Score for allergic rhinitis symptoms; Figure S1: Symptom-by-symptom plot of association between air pollutants and allergic rhinitis symptom scores in patients with allergic rhinitis; and Table S2: Lagged effects of air pollutants on allergic rhinitis symptom scores.
