# Supplementary material for: The Impact of Ambient Air Pollution on Allergic Rhinitis Symptoms: A Prospective Follow-Up Study
Source: Toxics. 2024 Sep 11;12(9):663. doi: 10.3390/toxics12090663 (PMC11436010; doi:10.3390/toxics12090663)
Supplement: Supplementary file 1 [file toxics-12-00663-s001.zip › toxics-3149727-supplementary.pdf]

Table S1. Classification of the severity of allergic rhinitis symptoms.

Symptom	Score
Sneezing<br>(Number of episodes of paroxysmal sneezing in a day)	0: 0~2;<br>1: 3~9;<br>2: 10~14;<br>3: ≥15.
Rhinorrhea<br>(Number of episodes of nose blowing in a day)	0: 0;<br>1: ≤4;<br>2: 5~9;<br>3: ≥10.
Nasal blockage	0: Never;<br>1: Few troubles;<br>2: Always;<br>3: Breathe through mouth almost all day.
Itchy nose	0: Never;<br>1: Every once in a while;<br>2: Tolerable;<br>3: Unbearable.
Itchy eyes	0: Never;<br>1: Slight;<br>2: Moderate;<br>3: Severe.

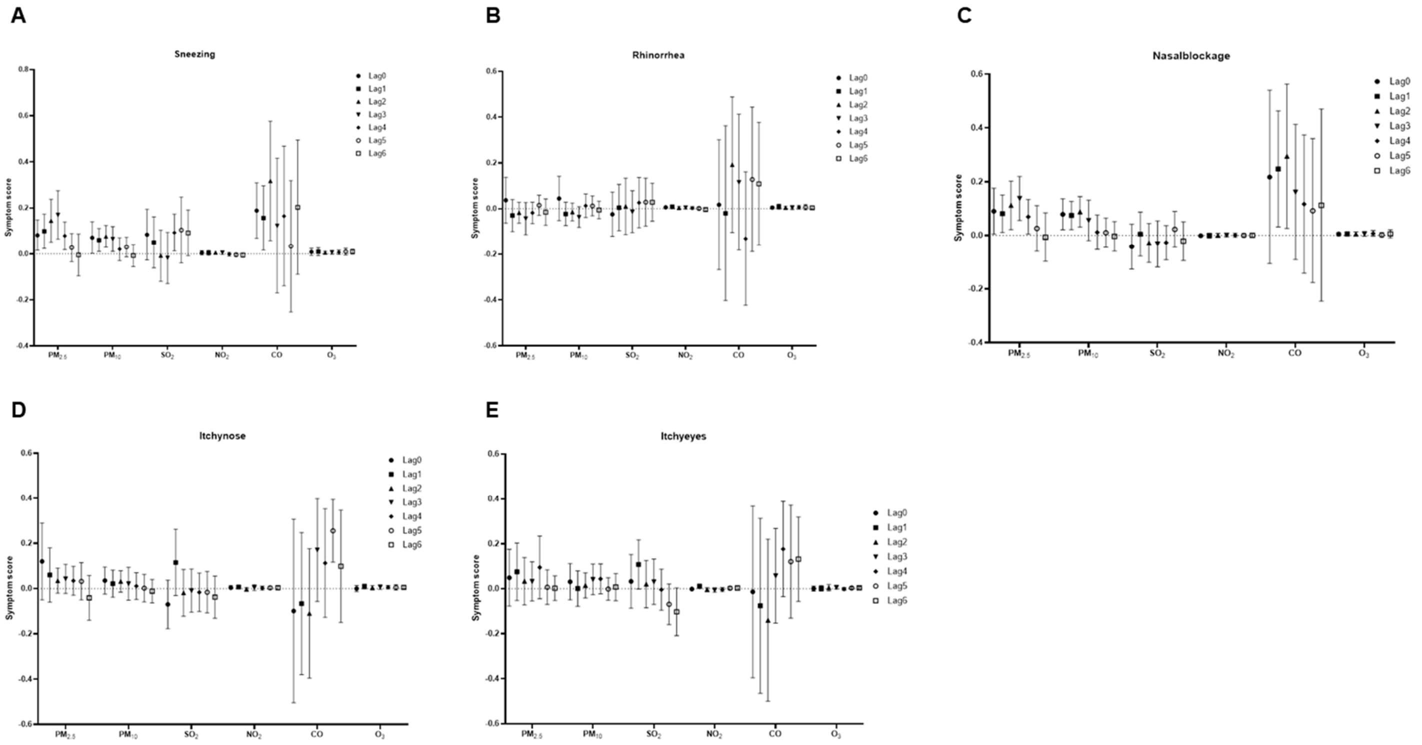

Figure S1. Symptom-by-symptom plot of association between air pollutants and allergic rhinitis symptom scores in patients with allergic rhinitis. $PM_{2.5}$, particulate matter with diameter <2.5 μm; PM_{10}, particulate matter with diameter <10 μm; SO_2, sulfur dioxide; NO_2, nitrogen dioxide; O_3, ozone; CO, carbon monoxide.

Table S2. Lagged effects of air pollutants on allergic rhinitis symptom scores.

	Sneezing	Rhinorrhea	Nasal blockage	Itchy nose	Itchy eyes
**$PM_{2.5}$**					
Log0	**0.075(0.019, 0.15), 0.020[a]**	0.014(-0.050, 0.147), 0.700	**0.081(0.010, 0.180), 0.007 [a]**	0.120(-0.051, 0.290), 0.606	0.039(-0.072, 0.180), 0.925
Log1	**0.081(0.034, 0.18), 0.002[a]**	-0.040(-0.095, 0.045), 0.103	**0.075(0.015, 0.154), 0.019 [a]**	0.093(-0.073, 0.160), 0.883	0.025(-0.019, 0.220), 0.684
Log2	**0.123(0.064, 0.246), <0.001 [a]**	-0.024(-0.060, 0.031), 0.322	**0.108(0.024, 0.204), 0.015 [a]**	0.024(-0.014, 0.095), 0.439	0.028(-0.069, 0.141), 0.492
Log3	**0.141(0.082, 0.286), 0.019 [a]**	-0.051(-0.110, 0.031), 0.498	**0.123(0.065, 0.226), 0.006 [a]**	0.035(-0.016, 0.111), 0.541	0.022(-0.049, 0.124), 0.763
Log4	**0.072(0.022, 0.142), 0.041 [a]**	-0.025(-0.061, 0.032), 0.443	**0.068(0.006, 0.135), 0.034 [a]**	0.029(-0.026, 0.101), 0.652	0.050(-0.017, 0.251), 0.280
Log5	0.030(-0.035, 0.087), 0.427	0.022(-0.032, 0.055), 0.853	0.040 (-0.065, 0.104), 0.711	0.014(-0.040, 0.122), 0.232	0.001(-0.067, 0.086), 0.864
Log6	-0.015(-0.091, 0.091), 0.436	-0.026(-0.066, 0.047), 0.852	-0.038(-0.076, 0.095), 0.673	-0.021(-0.148, 0.046), 0.738	-0.015(-0.042, 0.063), 0.145
**PM_{10}**					
Log0	**0.070(0.002, 0.140), 0.035 [a]**	0.024(-0.040, 0.150), 0.901	**0.074(0.015, 0.145), 0.024 [a]**	0.031(-0.022, 0.097), 0.328	0.010(-0.037, 0.120), 0.911
Log1	**0.060(0.010, 0.110), 0.009 [a]**	-0.030(-0.070, 0.032), 0.570	**0.071(0.024, 0.220), 0.006 [a]**	0.011(-0.032, 0.086), 0.596	-0.015(-0.069, 0.086), 0.745
Log2	**0.082(0.026, 0.120), 0.020 [a]**	-0.020(-0.050, 0.027), 0.324	**0.081(0.035, 0.240), 0.002 [a]**	0.034(-0.017, 0.078), 0.149	0.024(-0.045, 0.065), 0.935
Log3	**0.052(0.018, 0.124), 0.045 [a]**	-0.040(-0.080, 0.010), 0.143	0.034(-0.006, 0.140), 0. 077	0.035(-0.057, 0.087), 0.961	0.041(-0.027, 0.109), 0.794
Log4	0.021(-0.030, 0.070), 0.390	0.020(-0.042, 0.061), 0.241	0.014(-0.053, 0.075), 0.593	0.015(-0.050, 0.068), 0.853	0.042(-0.022, 0.111), 0.192

Log5	0.031(-0.014, 0.072), 0.202	0.015(-0.032, 0.054), 0.914	0.010(-0.044, 0.065), 0.675	0.020(-0.066, 0.052), 0.303	-0.011(-0.046, 0.053), 0.745
Log6	-0.014(-0.052, 0.042), 0.741	-0.011(-0.041, 0.035), 0.729	-0.019(-0.049, 0.058), 0.950	-0.025(-0.055, 0.045), 0.935	0.004(-0.051, 0.069), 0.110
**SO_2**					
Log0	0.084(-0.270, 0.194), 0.139	-0.038(-0.113, 0.078), 0.327	-0.042(-0.126, 0.042), 0.330	-0.070(-0.177, 0.037), 0.201	0.033(-0.087, 0.152), 0.593
Log1	0.050(-0.061, 0.161), 0.380	-0.009(-0.089, 0.112), 0.587	0.005(-0.077, 0.087), 0.906	0.144(-0.043, 0.245), 0.065	0.108(-0.001,0.217), 0.052
Log2	0.025(-0.133, 0.083), 0.650	-0.023(-0.093, 0.146), 0.440	-0.028(-0.101, 0.044), 0.445	-0.019(-0.122, 0.084), 0.720	0.020(-0.085, 0.125), 0.711
Log3	0.017(-0.142, 0.072), 0.882	-0.060(-0.072, 0.092), 0.865	-0.044(-0.111, 0.060), 0.258	-0.009(-0.104, 0.086), 0.847	0.031(-0.070, 0.132), 0.546
Log4	**0.093(0.012, 0.173), 0.025** [a]	-0.007(-0.063, 0.149), 0.802	-0.030(-0.089, 0.038), 0.331	-0.016(-0.101, 0.068), 0.705	-0.005(-0.095, 0.087), 0.926
Log5	0.140(-0.054, 0.226), 0.601	0.023(-0.073, 0.136), 0.524	0.023(-0.044, 0.090), 0.496	-0.016(-0.107, 0.075), 0.734	-0.069(-0.160, 0.021), 0.133
Log6	0.091(-0.009, 0.191), 0.074	0.044(-0.061, 0.102), 0.214	-0.021(-0.094, 0.051), 0.568	-0.038(-0.131, 0.055), 0.426	-0.106(-0.207, 0.005), 0.070
**NO_2**					
Log0	0.004(-0.005, 0.013), 0.408	**0.006(0.0001, 0.012) 0.048** [a]	-0.001(-0.008, 0.006), 0.853	0.004(-0.005, 0.013), 0.346	-0.002(-0.010, 0.008), 0.781
Log1	0.005(-0.007, 0.014), 0.464	**0.008(0.001, 0.016), 0.030** [a]	-0.001(-0.009, 0.010), 0.891	**0.005(0.001, 0.014), 0.031** [a]	**0.011(0.001, 0.021), 0.047** [a]
Log2	0.005(-0.003, 0.013), 0.224	0.004(-0.002, 0.010), 0.175	0.003(-0.007, 0.007), 0.885	-0.002(-0.011, 0.003), 0.557	-0.004(-0.012, 0.004), 0.338
Log3	0.005(-0.004, 0.012), 0.333	0.005(-0.001, 0.011), 0.091	0.002(-0.005, 0.008), 0.820	0.001(-0.007, 0.019), 0.825	-0.006(-0.015, 0.003), 0.195

Log4	-0.002(-0.010, 0.006), 0.595	0.003(-0.003, 0.009), 0.361	0.001(-0.0056, 0.009), 0.656	0.002(-0.006, 0.011), 0.595	-0.004(-0.012, 0.005), 0.402
Log5	-0.005(-0.012, 0.003), 0.221	0.003(-0.006, 0.007), 0.930	0.001(-0.006, 0.007), 0.682	0.003(-0.004, 0.011), 0.039	0.003(-0.005, 0.010), 0.495
Log6	-0.006(-0.015, 0.003), 0.203	-0.004(-0.010, 0.003), 0.276	0.001(-0.006, 0.007), 0.830	0.003(-0.005, 0.012), 0.437	0.003(-0.007, 0.013), 0.565
**CO**					
Log0	**0.199(0.063, 0.303), 0.021** [a]	-0.068(-0.214, 0.178), 0.085	0.188(-0.090, 0.554), 0.053	0.074(-0.562, 0.191), 0.081	0.102(-1.440, 0.297), 0.197
Log1	**0.122(0.037, 0.310), 0.007** [a]	-0.106(-0.352, 0.139), 0.352	**0.207(0.055, 0.481), 0.022** [a]	-0.047(-0.390, 0.237), 0.282	-0.172(-0.408, 1.352), 0.293
Log2	**0.283(0.076, 0.591), 0.001** [a]	0.124(-0.064, 0.516), 0.704	**0.236(0.060, 0.588), 0.008** [a]	-0.110(-0.396, 0.176), 0.557	-0.180(-0.414, 0.274), 0.405
Log3	0.118(-0.168, 0.418), 0.089	0.049(-0.142, 0.440), 0.890	**0.101(0.032, 0.452), 0.032** [a]	0.137(-0.038, 0.412), 0.380	0.028(-0.137, 0.281), 0.961
Log4	0.206(-0.158, 0.445), 0.297	-0.198(-0.384, 0.188), 0.088	0.096(-0.130, 0.384), 0.282	0.146(-0.142, 0.334), 0.587	0.167 (-0.031, 0.393), 0.570
Log5	0.051(-0.261, 0.309), 0.087	-0.039(-0.070, 0.492), 0.666	0.052(-0.154, 0.378), 0.533	0.164(-0.187, 0.415), 0.402	0.139(-0.139, 0.362), 0.786
Log6	0.120(-0.039, 0.527), 0.942	0.142(-0.174, 0.358), 0.651	0.078(-0.227, 0.486), 0.699	0.032(-0.110, 0.373), 0.590	0.146(-0.063, 0.311), 0.088
**O_3**					
Log0	0.011(-0.009, 0.032), 0.161	0.006(-0.001, 0.010), 0.906	0.006(-0.001, 0.011), 0.870	-0.001(-0.003, 0.0014), 0.229	0.001(-0.001, 0.002), 0.399
Log1	0.014(-0.010, 0.033), 0.359	0.006(-0.001, 0021), 0.623	0.007(-0.002, 0.014), 0.571	0.009(-0.002, 0.020), 0.282	0.002(-0.001, 0.003), 0.076
Log2	0.005(-0.021, 0.011), 0.387	0.004(-0.001, 0.011), 0.188	0.004(-0.001, 0.014), 0.344	-0.001(-0.002, 0.014), 0.266	0.001(-0.008, 0.020), 0.072

Log3	0.006(-0.022, 0.014), 0.122	0.001(-0.001, 0.015), 0.407	0.004(-0.001, 0.016), 0.234	0.005(-0.002, 0.017), 0.380	0.006(-0.002, 0.013), 0.921
Log4	0.003(-0.001, 0.017), 0.080	0.005(-0.001, 0.015), 0.546	0.005(-0.001, 0.020), 0.350	0.007(-0.002, 0.013), 0.823	0.001(-0.008, 0.003), 0.151
Log5	0.012(-0.007, 0.022), 0.185	0.001 (-0.001, 0.020), 0.053	0.001(-0.005, 0.012), 0.059	-0.001(-0.002, 0.019), 0.411	0.003(-0.001, 0.008), 0.767
Log6	0.008(-0.001, 0.020), 0.840	0.001(-0.001, 0.012), 0.172	0.002(-0.006, 0.023), 0.239	0.008(-0.002, 0.010), 0.867	0.003(-0.002, 0.009), 0.916

Data are presented as β(95%CI), *P*-value.

[a] *P*-value < 0.05
